# Highly sensitive and rapid point-of-care testing for HIV-1 infection based on CRISPR-Cas13a system

**DOI:** 10.1186/s12879-023-08492-6

**Published:** 2023-09-25

**Authors:** Xiaohui Li, Bin Su, Lan Yang, Zhihua Kou, Hao Wu, Tong Zhang, Lifeng Liu, Yao Han, Mengwei Niu, Yansong Sun, Hao Li, Taiyi Jiang

**Affiliations:** 1 Beijing Key Laboratory for HIV/AIDS Research, Clinical and Research Center for Infectious Diseases, Beijing Youan Hospital, Capital Medical University, Beijing, 100069 China; 2 State Key Laboratory of Pathogens and Biosafety, Beijing Institute of Microbiology and Epidemiology, Beijing, 100071 China

**Keywords:** HIV-1, CRISPR-Cas13a, RT-RAA, Point-of-care testing

## Abstract

**Background:**

Human immunodeficiency virus type one (HIV-1) is the leading cause of acquired immunodeficiency syndrome (AIDS). AIDS remains a global public health concern but can be effectively suppressed by life-long administration of combination antiretroviral therapy. Early detection and diagnosis are two key strategies for the prevention and control of HIV/AIDS. Rapid and accurate point-of-care testing (POCT) provides critical tools for managing HIV-1 epidemic in high-risk areas and populations.

**Methods:**

In this study, a POCT for HIV-1 RNA was developed by CRISPR-Cas13a lateral flow strip combined with reverse transcriptase recombinase-aided amplification (RT-RAA) technology, the results can be directly observed by naked eyes.

**Results:**

Moreover, with the degenerate base-binding CRISPR-Cas13a system was introduced into the RT-RAA primer designing, the technology developed in this study can be used to test majority of HIV-1 RNA with limit of detection (LOD) 1 copy/μL, while no obvious cross-reaction with other pathogens. We evaluated this method for detecting HIV-1 RNA of clinical samples, the results showed that the sensitivity, specificity, positive predictive value (PPV), negative predictive value (NPV) and accuracy were 91.81% (85.03- 96.19%), 100% (92.60–100%), 100% (96.41–100%), 39.14% (25.59–54.60%) and 92.22% (86.89–95.88%), respectively. The lowest viral load detectable by this method was 112copies/mL.

**Conclusion:**

Above all, this method provides a point-of-care detection of HIV-1 RNA, which is stable, simple and with good sensitivity and specificity. This method has potential to be developed for promoting early diagnosis and treatment effect monitoring of HIV patients in clinical.

**Supplementary Information:**

The online version contains supplementary material available at 10.1186/s12879-023-08492-6.

## Background

With the widespread use of antiretroviral therapy (ART) after it was invented in the 1970s, the mortality and morbidity of HIV/AIDS population has been remarkably reduced and the survival time greatly extended, meanwhile most common complications of HIV infection have been controlled effectively. Recent data from the WHO estimates that the number of confirmed HIV patients reaches up to 37.7 million, of which 70% are from sub-Saharan Africa. In China, the number of HIV patients is increasing rapidly too [1, 2]. The major HIV-1 strain in China is CRF07_BC (accounted for 41.3%), followed by CRF01_AE (32.7%), CRF08_BC (11.3%) and subtype B (4.0%) [3, 4]. Many new approaches of HIV treatment have been confirmed effectively, but the researchers are still slowly getting closer to finding a true cure or useful vaccine to this disease, life-long antiviral treatment is required and necessary for AIDS/HIV patients at least in quite a long future [5–7]. So, the current strategy of AIDS/HIV prevention and control still focuses on stopping the virus transmission, and methods for accurate and quick detection are of great vital for the successful implementation of this strategy.

Methods for detecting HIV, including western blotting, enzyme-linked immunosorbent assay (ELISA) and polymerase chain reaction (PCR) have been widely used in hospitals and other medical institutions utilizing antibodies, antigens or DNA [8]. However, antibody- and antigen-based testing cannot confirm the “window period”, leading to false-negative results [8, 9]. Although PCR is regarded as the gold standard and main method for HIV early diagnosis, clinical treatment effect/drug resistance monitoring, but it relies on technologies and instruments too much and suffers from the primer designing, rigorous thermal cycling, and long assay time [8, 10]. In contrast, the quantitative analysis of HIV RNA is a reliable method owing to that it narrows the “window period” to 10 days.

The outbreak of Corona Virus Disease 2019 (COVID-19) has caused rapid development of new technologies for virus detection, including but not limited on RT-qPCR, gene sequencing and isothermal amplification [11]. CRISPR-Cas system (clustered regularly interspaced short palindromic repeat and CRISPR-associated protein) has raised much attention in nucleic acid detection for its higher sensitivity and specificity, including CRISPR-Cas 9a, CRISPR-Cas 12a and CRISPR-Cas 13a [12, 13]. Among them, it is reported that Zhang and colleagues combined RNA-guided, RNA-targeting CRISPR effector Cas13a with isothermal amplification and established a Cas13a-based molecular detection platform, termed SHERLOCK (Specific High Sensitivity Enzymatic Reporter UnLOCKing) to detect Zika and Dengue, this technology is combined or paired with other methods like DETECTR(DNA Endonuclease-Targeted CRISPR Trans Reporter), CDetection (Cas12b-mediated DNA detection) and others to detect viruses, bacteria and other pathogenic agents [14–17]. However, the current CRISPR-Cas13a system needs instruments to show the results. It is reported that CRISPR-based nucleic acid detection can be combined with reverse transcription recombinase-aided amplification (RT-RAA) to enable rapid, accurate, and early detection of SARS-CoV-2 [18]. RT-RAA detection method can directly use RNA as a template for pathogen detection, amplification products can be detected by using portable instruments, such as lateral flow strips and portable blue light instruments [19].

Based on the ERASE (Easy-Readout and Sensitive Enhanced) lateral flow strip assay for COVID-19 detection established by our collaborator [20], we took advantages of this well-established technology for detecting HIV-1 RNA, experiments from multiple angles confirmed that it could offer excellent or at least the same sensitivity and specificity compared with other conventional detection methods. So here in this study, we report this lateral flow strip for simple, and accurate detection of HIV-1 RNA via the assistance of CRISPR-Cas13a, showing great potential for HIV detection in self-testing and clinical applications.

## Materials and methods

### Clinical samples

Whole blood samples of 110 HIV-1-infected patients and 48 healthy individuals were collected during Jan to May of 2022 by the Beijing Youan Hospital, Capital Medical University. All the 110 HIV-1-infected patients were ART naïve when diagnosis. Patients who had virologic failure were excluded. The whole blood samples were centrifuged to collect plasma, the collected plasma RNA was extracted by the RNA extraction kit according to the manufacturer’s protocol and then stored at -80℃.

### Reagents and instruments

RT-RAA amplification kit was brought from Hangzhou ZC Bio-Sci&Tech (Shanghai, China). T7-RNA polymerase, RNase inhibitor, T7 transcription kit and NTP mix were purchased from New England Biolabs (Ipswich, MA, USA). Cas13a protein was from Jiang Shen Biotechnology (Ningbo, China). Fluorescent report RNA was purchased from Thermo Fisher company (MA, USA). RT-PCR kit was brought from Takara Bio (Japan) and 2 × Super PFX master mix was from Cowin Biotech (Jiangsu, China), QIAamp®Viral RNA mini kit was from Qiagen (Germany). *RT-qPCR HIV-1 reaction kit was from* DaAn Gene Co., Ltd. of Sun Yat-sen University (Guangdong, China). Main instruments used in this study included Applied Biosystems Veriti PCR (Thermo Fisher, USA) and CFX 96 Touch Real-Time PCR Detection System (Bio-Rad, USA).

### Nucleic acid preparation

The RT-RAA primer was designed according to the reported work [15], T7 promoter sequence was added to the 5’ end of the forward primer. Four crRNA candidates were developed from the conserved regions between the upstream and downstream primers according to the crRNA designing principles. Primers for RT-RAA of HIV was designed by the Primer 3 plus software with compatible crRNAs. crRNA sequence was synthesized by Tian Yi Hui Yuan Biotechnology (Beijing, China) (Supplementary Tables 1 and 2). The PCR reaction components included 25μL 2 × Super Pfx MasterMix, 2μL Primer, 2μL template RNA, 2μL (10 nM) forward and reverse primers, the reaction mixture was assembled with enzyme-free water into 50μL volume in a thin walled 0.2 mL PCR tube. PCR temperature cycle was set at 95℃ for 5 min, followed by 35 cycles at 95℃ for 30 s, 55℃ for 30 s, 72℃ for 15 s, with a final extension at 72℃ for 10 min. The PCR products were purified by T7 transcription kit and kept in the -80℃ freezer. The HIV-1 RNA templates in this study were synthesized, the HIV-1 (standard strain GenBank ID: k03455.1) plasmid containing a fragment of 273 bp was constructed by Psmart-LC (KanR) plasmid backbone and then transcribed to HIV-1 RNA, the HIV-1 RNA were stored at -80 ℃ after gradient dilution. Formula: DNA/RNA copy number = {[6.02 × 10^23^ × plasmid concentration (ng/mL) × 10^–9^]} / [DNA/RNAlength × 660/340].

### RT-RAA amplification

The RT-RAA amplification was performed according to the manufacturer’s instruction in a final volume of 50μL containing 33.5μL Buffer A, 2μL Primers with the final concentration of 10 nmol/L, 10μL template and 2.5μL Buffer B. After mixing and centrifuging, the reaction was performed at 42℃ for 30 min. The amplified products were then electrophoresed on a 1.5% agarose gel and stored at 4℃ after purification for CRISPR detection.

### Fluorescence CRISPR assay

The fluorescence CRISPR assay was developed by the reported work [21]. CRISPR-Cas13a strategy contained 5μL RT-RAA amplification products, 1μL LwCas13a protein (25 nmol/L), 2μL NTP Mix (2.5 nmol/L), 0.75μL T7 RNA polymerase, 2μL RNase inhibitors, 0.25μL magnesium chloride solution (10 mmol/L), 0.5μL HEPES buffer (20 mmol/L), 1.5μL crRNA (2 μmol/L), 2.5μL fluorescence reporter-based RNA (2 nmol/L) and 9.75μL DEPC-treated water. The final products of this strategy were measured by PCR under excitation wavelength of 490 nm and emission wavelength of 520 nm, the fluorescence signals of reaction at 37℃were recorded every 2 min for 35 times.

### Lateral-flow strip CRISPR assay

The reaction system of the lateral-flow strip CRISPR assay contained 5μL RT-RAA amplification products, 4μL NTP Mix (2.5 mmol/L), 2μL RNase inhibitors, 1μL T7 RNA polymerase, 0.5μL magnesium chloride solution (10 mmol/L), 1μL HEPES buffer (20 mmol/L), 3μL crRNA (2 μmol/L), 5μL reporter RNA (nmol/L) and 26.5μL DEPC-treated water. After mixing and centrifuging softly, the reaction was performed at 37℃for 30 min and the products were sprayed onto the CRISPR test paper and results were visualized after 2 min.

### RT-qPCR assay

The RT-qPCR assay was performed by the manufacturer’s instruction. Reaction cycle parameters were set as reverse transcription at 50 °C for 15 min, denaturation at 95 °C for 15 min, followed by 45 cycles of amplification, 94 °C for 15 s and 55 °C for 45 s.

### RT-PCR assay

The RT-PCR assay was performed as the manufacturer’s instruction. The reaction liquid was composed of 5μL extracted RNA, 25μL Ex Taq Mix, 1μL upstream and downstream primers with final concentration of 200 nmol/L and 18μL ddH_2_O. The following temperature cycle was used: 95 °C for 5 min, followed by 35 cycles at 95 °C for 10 s, 70 °C for 30 s, and 72 °C for 1 min, with a final extension at 72 °C for 5 min. The reaction products were then electrophoresed on a 1.5% agarose gel for detection.

### Results analysis

The visualized of the lateral-flow strip CRISPR detection was followed the rule of “line elimination” method. The presence of Control line (C line) only correlated with a positive test result, the presence of both Control line and Test line (T line) correlated with a negative test result, the absence of Control line was referred as test failure and needed to redo with a new strip. SPSS20.0 software was hired to analyze the agreement between fluorescence CRISPR assay and Lateral-flow strip CRISPR assay, the k value of the Cohen’s kappa between 0.81–0.99 was considered as near perfect agreement.

## Results

### Selection of RT-RAA amplification primers and HIV-1 RNA nucleic acid target, development of ERASE detection method for HIV-1 RNA

**Fig. 1:**
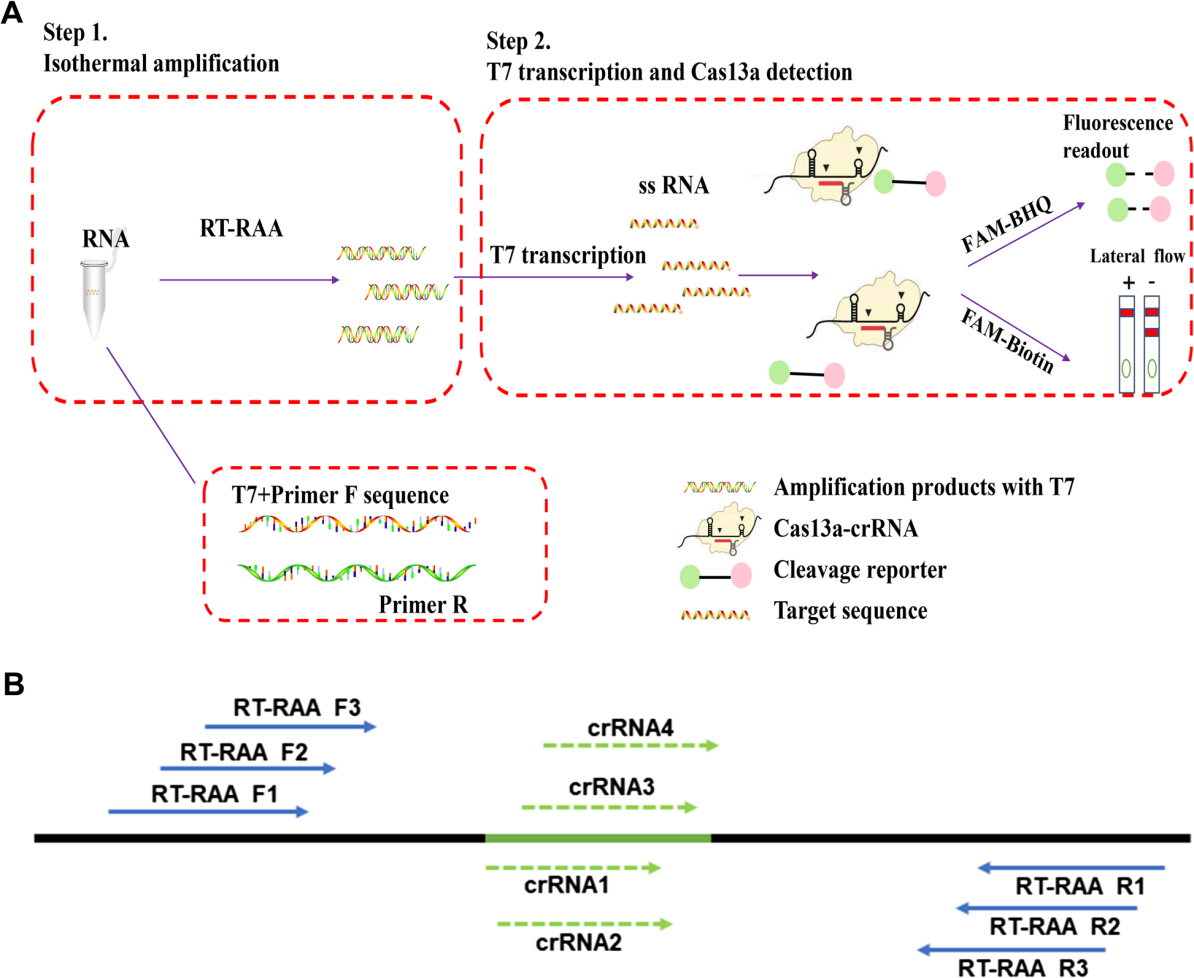
CRISPR-based latera-flow strip for HIV-1 RNA detection. **A** Schematic of the lateral flow strip for CRISPR-based detection. CRISPR-Cas13a is combined with RT-RAA, CRISPR-Cas13a detection is performed after RT-RAA and the results are read by fluorescence assay or side-flow strips. **B** Schematic of RT-RAA primer and crRNA design, RT-RAA upstream primer and downstream primer are combined in pairs

In this study, a rapid nucleic acid detection assay for HIV-1 RNA was developed based on the ERASE method for COVID-19 detection established by our collaborator (Fig. 1A). First, HIV-1 RNA extracted from plasma samples was amplified by the RT-RAA technology for 30 min, the amplified products were then added to the CRISPR-Cas13a system. Second, in the CIRSPR/Cas13a system, Cas13a-crRNA complex recognized the target RNA sequence specifically and activated the cleavage activity of Cas13a. Third, Cas13a sliced the surrounding reporter RNA nonspecifically, the results can be measured by a following fluorescence assay or side-flow strips directly.

To develop the RT-RAA-CRISPR-Cas13a assay for detecting HIV-1 RNA, 5086 complete genomic sequences of HIV-1 were downloaded from the HIV network database (http://www.hiv.lanl.gov, released in 2018) and then compared to the genomic sequences in MEGA7.0 and Jalview1.83, 9 pairs of RT-RAA primers and 4 crRNA were designed (Fig. 1B, Table S1). A highly conserved sequence (4620-4922 bp) were screened in the HIV pol region (Table S2) and the plasmid of a HIV-1 standard strain Psmart-LC (KanR) (GenBank ID: k03455.1) was constructed.

**Fig. 2:**
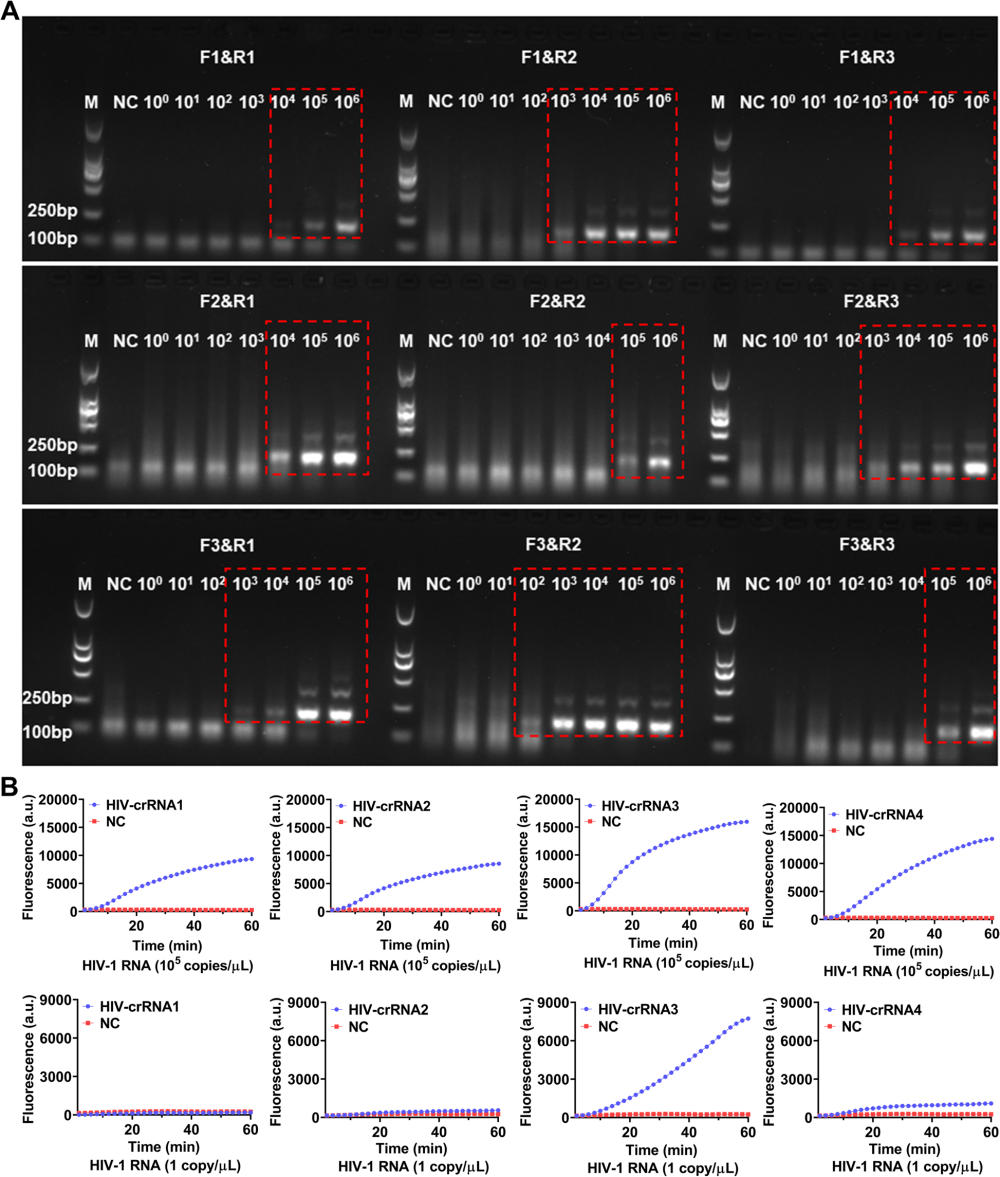
Screening of RT-RAA primers and crRNA. **A** The HIV-1 RNA template was diluted in gradient and amplified separately using 9 pairs of RT-RAA primers. The amplification products are shown in the red dashed boxes (The uncropped result is shown in Supplementary Figure S1). M refers to Marker, NC refers to ddH_2_O. **B** HIV-1 RNA templates (concentration of 1 × 10^5^ copies/μL and 1 × 10^1^ copies/μL) are used for RT-RAA amplification and detected by CRISPR-based fluorescence assay. M refers to Marker, NC refers to negative control

For screening the RT-RAA primers, a serial of diluted HIV-1 RNA templates (1 × 10^6^–1 × 10^0^ copies/μL) were used for RT-RAA amplification, the amplified products were then analyzed by electrophoresis. The results showed that all the 9 pairs of RT-RAA primers were able to amplify HIV-1 RNA by RT-RAA with the RNA template concentration at 1 × 10^6^ copies/μL, only F3&R2 primers amplified a weaker HIV-1 RNA band (179 bp) with the RNA template concentration was 1 × 10^2^ copies/μL, the other 8 pairs of RT-RAA primers failed to amplify any target RNA by RT-RAA (Fig. 2A). Therefore, F3&R2 primer were selected as the best RT-RAA primers for this study. Next, HIV-1 RNA templates with concentration of 10^5^ copies/μL and 1 copy/μL were used for RT-RAA amplification respectively and the amplified products were then detected by CRISPR-based fluorescence assay for screening crRNA.

The results showed that when the template RNA concentration was 10^5^ copies/μL, after 1 h, the average fluoresence intensity of the 4 crRNA reactions, which were 103.00 ± 80.42 (a.u.), 8296.00 ± 131.10 (a.u.), 15701.00 ± 478.90 (a.u.) and 14145.00 ± 356.80 (a.u.) respectively were significant higher than the negative control 259.33 (a.u.) (Fig. 2B). When the template RNA concentration was 1 copy/μL, after 1 h, the average fluoresence intensity of the 4 crRNA reactions were 64.00 ± 5.90 (a.u.), 306.30 ± 7.81 (a.u.), 7496.00 ± 37.68 (a.u.) and 801.30 ± 33.52 (a.u.), while the negative control was 246 ± 7.93 (a.u.) (Fig. 2B). Therefore, the crRNA-3 which had the highest fluorescence intensity was selected as the nucleic acid detection target for subsequent experiments.

### Sensitivity of the CRISPR-based lateral-flow strip assay for detecting HIV-1 RNA

**Fig. 3:**
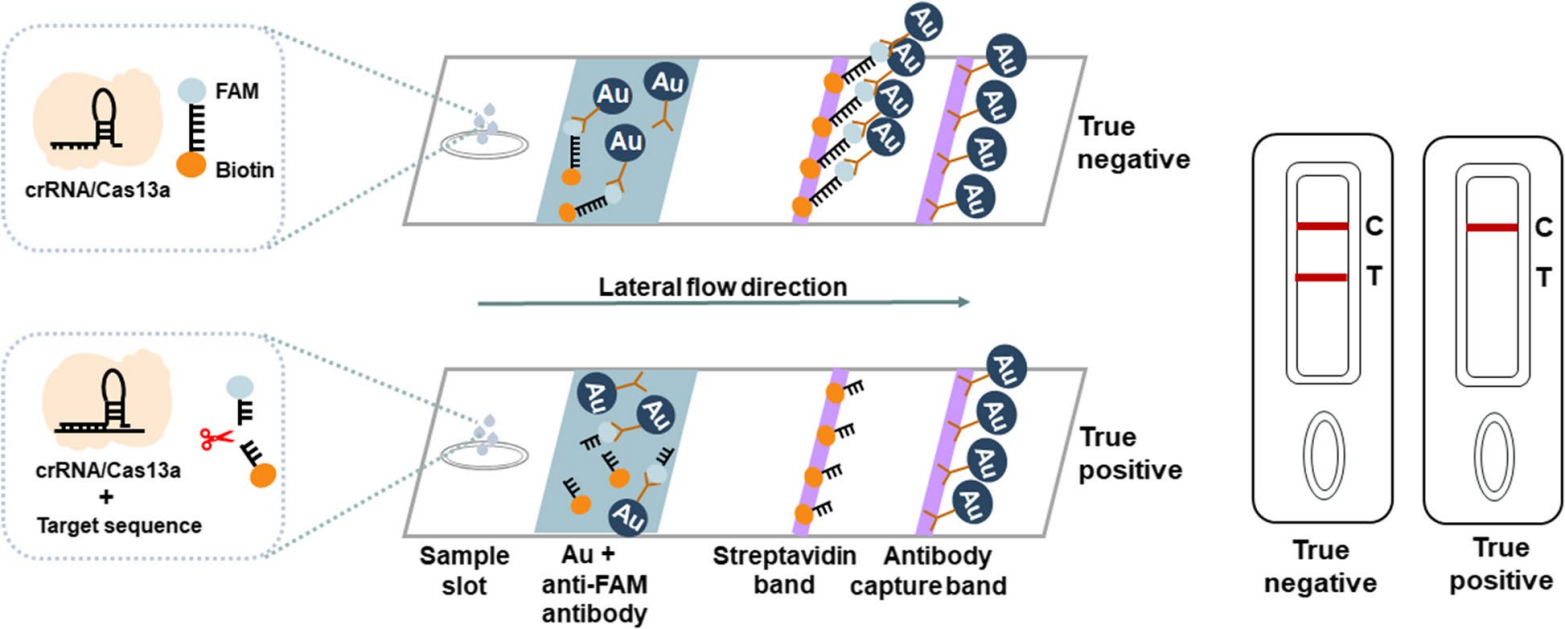
ERASE lateral flow strip for CRISPR-based detection. Schematic of the ERASE lateral flow strip for CRISPR-based detection. Gold-labeled FAM-biotinylated reporter molecules flow to the test capture band, and redundant gold nanoparticles flow to the control capture band. Upon recognition of the target RNA, the crRNA/Cas13a complex cleaves to the reporter molecule, allowing passage by the test band

**Fig. 4:**
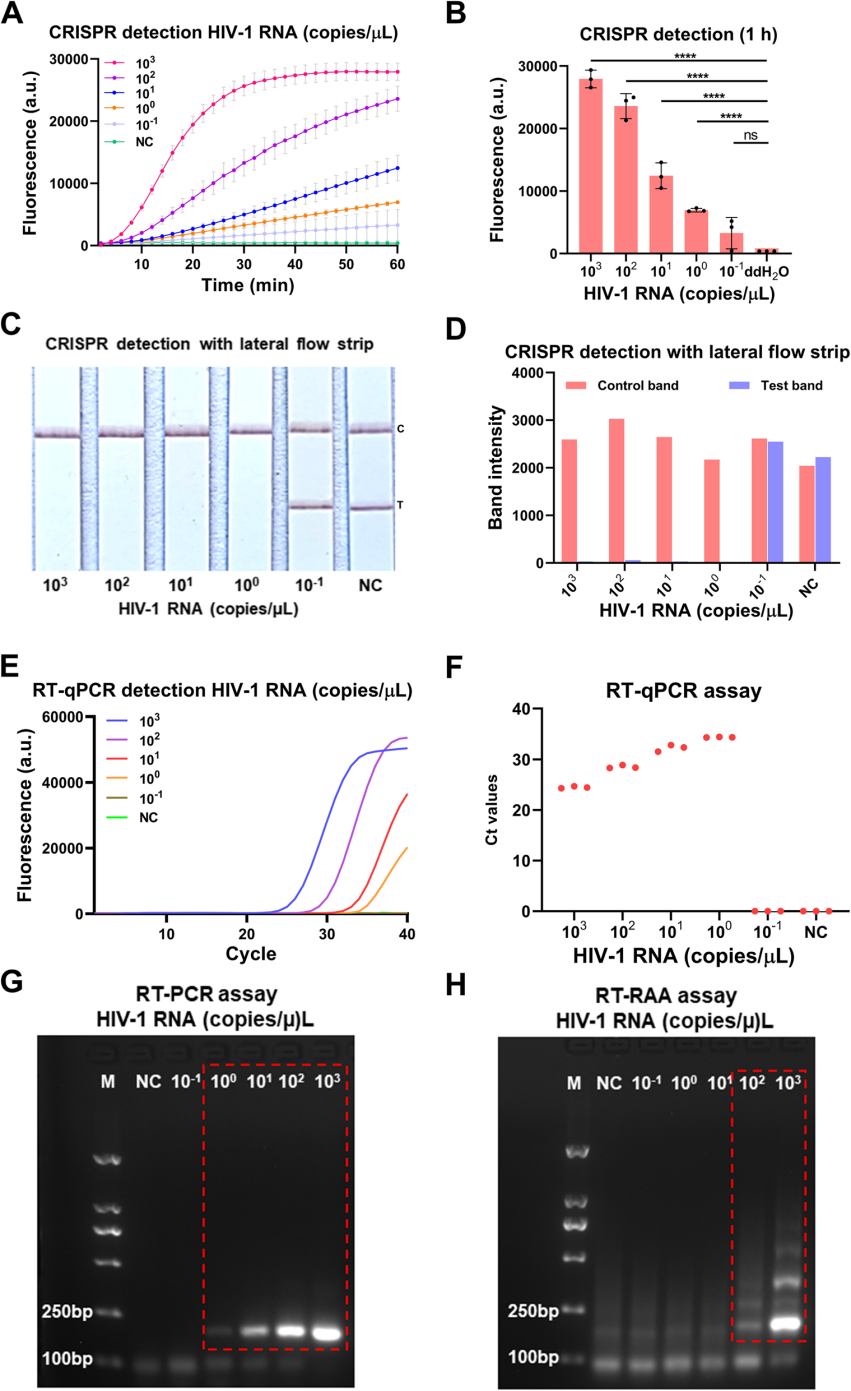
Evaluation of the CRISPR-based latera-flow strip for HIV-1 RNA detection. **A-B** CRISPR-based fluorescence assay for detecting HIV-1 RNA concentration at 1 h. **C-D** CRISPR-based latera-flow strip assay for HIV-1 RNA detection and the band intensities. **E–F** Distribution of Ct values of RT-qPCR method and amplification curve of HIV-1 RNA at different concentrations. **G** Agarose gel electrophoresis of HIV-1 RNA with different concentrations after RT-PCR (The uncropped result is shown in Supplementary Figure S3). **H** Agarose gel electrophoresis of HIV-1 RNA with different concentrations after RT-RAA (The uncropped result is shown in Supplementary Figure S3). NC: Negative control, "****" means *P* < 0.0001, "***", "**" means *P* < 0.005, and "ns" means no significant difference. All experiments were carried out in triplicate for data analysis

To evaluate the sensitivity of the our developed CRISPR-based lateral-flow strip assay for detecting HIV-1 RNA (Fig. 3), a serially diluted HIV-1 RNA templates (1 × 10^3^–1 × 10^–1^ copies/μL) were measured by CRISPR-based fluorescence assay, CRISPR-based lateral-flow strip assay, RT-qPCR, RT-PCR and RT-RAA amplification. The results showed that the sensitivity of the CRISPR-based fluorescence assay reached 1 copy/μL in 10 min (Supplementary Figure S2) and the limit of detection (LOD) was 1 × 10^–1^ copies/μL at 1 h (detected twice in 3 independent repeated trials) (Fig. 4A and B). CRISPR-based lateral-flow strip assay results indicated that when the template RNA concentration was 1 copy/μL, only the C line can be captured but the T line faded away in 2 min, this was recognized as a positive result. When the template RNA concentration was 1 × 10^–1^ copies/μL, both C line and T line can be visualized, this was recognized as a negative result. So, the sensitivity of CRISPR-based lateral-flow strip assay for detecting HIV-1 RNA was 1 copy/μL (Fig. 4C and D). Similar results were found using RT-qPCR and RT-PCR, both of them were able to steadily detect the template RNA at concentration of 1 copy/μL (Fig. 4E-G). The LOD of the RT-RAA strategy was 1 × 10^2^copies /μL for HIV-1 RNA detection (Fig. 4H).

The results of comparing the sensitivity of CRISPR-based lateral-flow strip and CRISPR-based fluorescence assay were shown in Fig. 4A-D. The fluorescence intensity of the HIV-1 RNA with concentration of 1 × 10^3^, 1 × 10^2^, 1 × 10^1^, 1 × 10^0^ and 1 × 10^–1^ copies/μL all increased as the reaction time lapsed, after 40 min, the average fluorescence intensity of all the HIV-1 RNA reactions with different concentration mentioned above, which were 26921 ± 827.3 (a.u.), 17107 ± 1111 (a.u.), 7031 ± 744.1 (a.u.), 4115 ± 250.0 (a.u.) and 1780 ± 913.3 (a.u.), were significant higher than the negative control which was 450.00 ± 1.00 (a.u.), no obvious fluorescence signals were detected in the group with the target RNA concentration of 1 × 10^–1^ copies/μL and the negative control group. It indicated that the CRISPR-based lateral-flow strip assay and CRISPR-based fluorescence assay shared the same sensitivity when detecting HIV-1 RNA.

The serially diluted HIV-1 RNA templates (1 × 10^3^–1 × 10^–1^ copies/μL) were used to evaluate the sensitivity of RT-qPCR, RT-PCR and RT-RAA amplification for detecting HIV RNA (Fig. 4E-H), the sensitivity of the RT-qPCR was 1 × 10^0^ copies/μL and the LOD of the RT-RAA amplification assay was 1 × 10^2^ copies/μL. The results showed that the sensitivity of CRISPR-based lateral-flow strip assay was consistent with the RT-PCR and RT-qPCR, but higher than the RT-RAA amplification assay.

### The specificity evaluation of CRISPR-based lateral-flow strip assay for detecting HIV-1 RNA

**Fig. 5:**
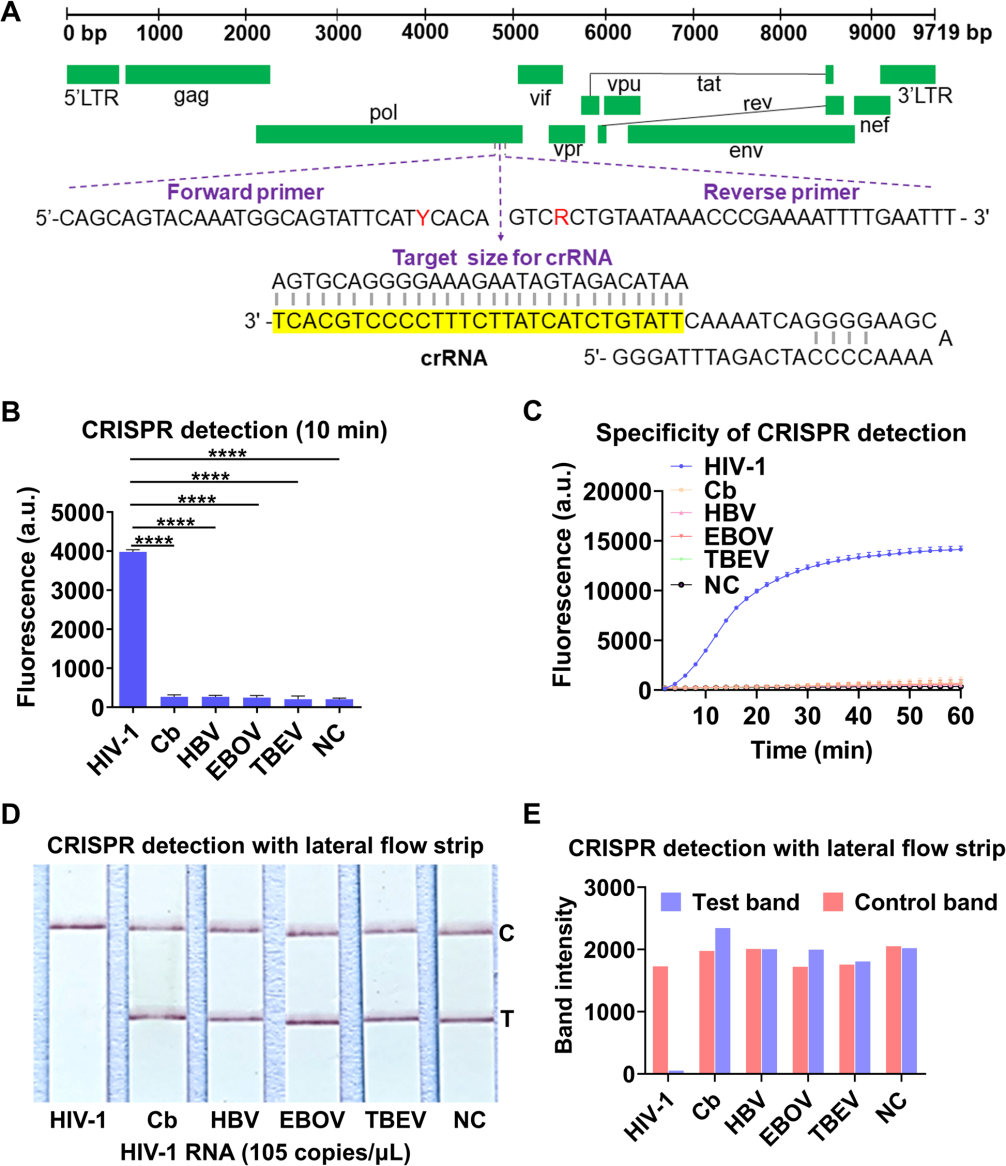
Evaluation of the specificity of CRISPR-Cas13a for HIV-1 detection. **A** The genome map shows the site of the target crRNA and primers on the HIV-1 pol gene. RT-RAA primers have degenerate bases R (A/G) and Y (C/T), which are marked in red; the crRNA sequence is marked in yellow. **B** Fluorescence method CRISPR-Cas13a detects 5 kinds of pathogen nucleic acids. **C** Fluorescence value of different pathogens when CRISPR-Cas13a is detected for 10 min by fluorescence method. **D** Test strip method CRISPR-Cas13a to detect nucleic acids of different pathogens. **E** Band intensities of each test strip were quantified using ImageJ. C: Control line, T: Test line, NC: Negative control, "****" means *P* < 0.0001, the above experiments were performed 3 independent repeat experiments

In order to detect the major HIV-1 strains worldwide by the CRISPR-Cas13a system, degenerate primers of RT-RAA and crRNA was introduced into the HIV-1 pol region (Fig. 5A) to improve the specificity of the method. Then four pathogens including Coxiella burnetiid (Cb), Hepatitis B virus (HBV), Ebola virus (EBOV) and Tick-borne encephalitis virus (TBEV) were compared with HIV-1 by CRISPR-based fluorescence assay and CRISPR-based lateral-flow strip assay in this study, the results of CRISPR-based fluorescence assay showed that the fluorescence intensity of HIV-1 reached 3773.00 ± 62.02 (a.u.) after 10 min, it was significantly higher than the fluorescence intensity of the nucleic acids of other four pathogens (*P*˂0.05) (Fig. 5B). The fluorescence intensity of HIV-1 detection was enhanced quantitatively and stabilized after 1 h, and no significant cross-reaction with other pathogens was observed (Fig. 5C). The CRISPR-based lateral-flow strip assay showed that only the HIV-1 RNA detection was positive (T line faded away and C line appeared), the other four pathogens were all negative (Both C line and T line can be observed) (Fig. 5D and E).

### Evaluation of the CRISPR-based lateral-flow strip assay by clinical samples

**Fig. 6:**
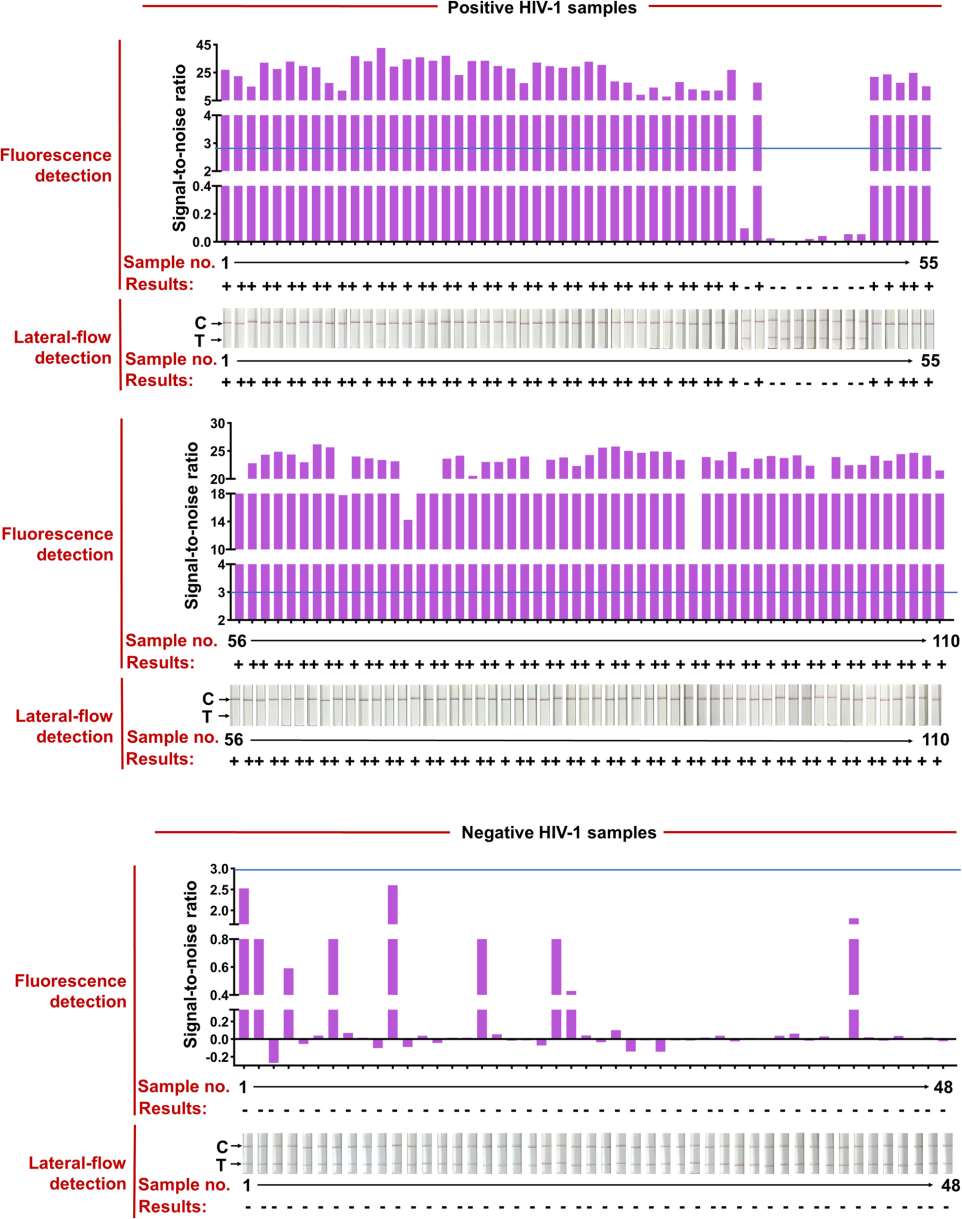
RT-RAA-CRISPR-Cas13a detected HIV-1 in 158 plasma clinical samples. Positive HIV-1 clinical samples were classified according to their C_t_ values from RT-qPCR and the total number of clinical samples in each Ct classification is shown. Representative lateral-flow strip and fluorescence detection of each C_t_ bin as well as HIV-1 negative samples are shown. The full dataset is shown in Supplementary Fig. 1. For lateral-flow strip readings, the disappearance of the colored test strip, often combined with the appearance of the colored control strip, indicates a positive HIV-1 result. For fluorescence readings, we set a threshold for the signal-to-noise (S/N) ratio of fluorescence intensity (blue line) (noise is the fluorescence intensity from negative samples performed in parallel with water as input), and positive results are 3. Bottom row shows the number of false negative samples; false negative result rates are shown for each C_t_ bin and for the two RT-RAA-CRISPR-Cas13a readout modes

The HIV test kits, RT-qPCR HIV-1 reaction kit, used for clinical samples were purchased from the DaAn Gene Co., Ltd. of Sun Yat-sen University. 158 clinical samples from 110 HIV-1-positive patients (Ct values varied from 20 to 40) and 48 HIV-1-negative individuals were used to evaluate the effectiveness of the CRISPR-based lateral-flow strip assay developed in this study (Fig. 6 and Table 1). All the clinical samples were detected by the CRISPR-based fluorescence assay and lateral-flow strip CRISPR assay respectively (Supplementary Figure S4).

**Table 1:** CIRSPR-Cas13a lateral flow strips to detect Ct values in clinical samples

Ct Value	CRISPR		Positive rate	Total
	Positive	Negative		
< 25	11	0	100%	11
25 ~ 30	50	0	100%	50
30 ~ 35	35	0	100%	35
35 ~ 40	5	9	35.71%	14
Total	101	9		110

Compared with RT-qPCR, the positive rate of CRISPR-based lateral-flow strip assay for detecting HIV-1 RNA was 91.81% (101/110) and the negative rate was 100% (48/48), the overall clinical compliance rate was 94.3% with the Kappa value of 0.872 (*P* < 0.001), so as the detection results of the CRISPR-based fluorescence assay (Fig. 6, Supplementary Figure S4 and Table 2). The Ct value of HIV-1 RNA from clinical samples reached 37.32 (112 copies/mL) detected by the CRISPR-based lateral-flow strip assay (Fig. 6, Supplementary S5). For samples with Ct value less the 37.32 (viral load more the 112 copies/mL), the positive rate was 100% (100/100) and negative rate was 100% (100/100), and for samples with Ct value higher than 37.32 (viral load more the 112 copies/mL), the positive rate was 10% (1/10) and negative rate was 100% (48/48) (Fig. 6 and Supplementary S5). Briefly, the results showed that the LOD for HIV-1 RNA by the CRISPR-based lateral-flow strip assay was 112 copies/mL, which was highly consistent with the clinical evaluation.

**Table 2:** The detection of clinical samples by CIRSPR-Cas13a lateral flow strip

	**RT-qPCR**		**Sensitivity**	**Specificity**	**PPV**	**NPV**	**Accuracy**
	**Positive**	**Negative**	**(95% CI)**	**(95% CI)**	**(95% CI)**	**(95% CI)**	**(95% CI)**
**CRISPR-Fluorescence**							
**Positive**	101	0	91.81%	100%	100%	39.14%	92.22%
**Negative**	9	48	(85.03–96.19%)	(92.60–100%)	(96.41–100%)	(25.59–54.60%)	(86.89–95.88%)
**Total**	110	48					
**CRISPR-strip**							
**Positive**	101	0	91.81%	100%	100%	39.14%	92.22%
**Negative**	9	48	(85.03–96.19%)	(92.60–100%)	(96.41–100%)	(25.59–54.60%)	(86.89–95.88%)
**Total**	110	48					

The gold standard for HIV is RT-qPCR; *PPV* Positive Predictive Value, *NPV* Negative Predictive value, *CI* confidence interval

In order to evaluate the effect of the CRISPR-based lateral-flow strip assay for detecting different HIV-1 subtypes, 60 clinical samples covering three major HIV-1 subtypes in China: subtype 01_AE, 07_BC and subtype B were collected (20 samples of each subtype) and specific crRNAs were designed for each subtype (Supplementary Table S3). The HIV-1 RNA was detected by CRISPR-based lateral-flow strip assay and compared with RT-qPCR and CRISPR-based fluorescence assay, the results showed all the 60 HIV-1 positive samples were tested positive by CRISPR-based lateral-flow strip assay, as well as by the CRISPR-based fluorescence assay and RT-qPCR (Table 3, Supplementary Figure S5).

**Table 3:** Concordance between RT-qPCR and CRISPR detection of clinical samples

	**HIV-1 different subtypes**			
	**CRF01_AE**		**CRF07_BC**	**B**
**Fluorescence CRISPR readout**	**Positive**	20/20 (100%)	20/20 (100%)	20/20 (100%)
	**Negative**	0	0	0
	**Total**	20	20	20
**Lateral-flow strip CRISPR readout**	**Positive**	20/20 (100%)	20/20 (100%)	20/20 (100%)
	**Negative**	0	0	0
	**Total**	20	20	20

## Discussion

Early detection and effective treatment are two important strategies for HIV prevention and control. Optimization of current HIV detection technology can help to improve the effectiveness of HIV diagnosis and treatment. In this study, we developed an on-site nucleic acid test based on CRISPR technology, which allowed naked eye for HIV-1 detection by the following lateral-flow strip, this technique has been preliminarily proved to be very sensitive and not lab-dependent, we referred it as CRISPR-based lateral-flow strip assay. This assay has the potential to enable a fast, accurate, and private option for point-of-care HIV-1 nucleic acid detection.

Current HIV/AIDS diagnosis and treatment have been put forward higher requirements to HIV detection technology. First, methods for detecting HIV-1, including western blotting, ELISA and PCR have been widely used in hospitals and other medical institutions utilizing antibodies, antigens or DNA [8]. However, antibody- and antigen-based testing cannot confirm the “window period”, leading to false-negative results [8, 9]. PCR relies on technologies and instruments a lot. There was a team used self-digitization through automated membrane-based partitioning (STAMP) method to digitalize the CRISPR-Cas13 assay (dCRISPR), this method could quantitatively detect HIV RNA samples within a certain concentration range, with LoD of 2 copies/μL [22]. Although this method can achieve quantitative detection for RNA samples without amplification, it has certain limitation for early screening because negative results are likely to occur when detecting low viral load samples (Ct > 32). Another team developed a nucleic acid amplification-free method called the AuNPs-tagging based CRISPR-Cas12a bioassay platform, the LoD for dectecting HIV plasmid was 1.05aM, the signal amplification was realized by integrating the self-amplification effect of CRISPR-Cas12a with the enhancement effect of the large number of detectable atoms inside each gold nanoparticle. The whole detection took only 40 min, but only carried out in the HIV plasmid instead of HIV RNA and clinical samples [23]. There were some other studies hired RT-RAA combined CRISPR-Cas12a technology to detect HIV, with Lod of 20 copies/μL for DNA and RNA samples and 123copies/mL for clinical samples [24]. This method had a better sensitivity for detecting clinical samples, but took longer detection time and a fluorescence reader was required for reading the results. In the future, this technology has potential to became a POCT product by shortens the detection time and optimizing the result reading method.

In contrast, the quantitative analysis of HIV RNA is a reliable method owing to that it narrows the “window period” to 10 days. Second, ART inhibits HIV replication but is not curative and must be taken for life, the treatment effect is affected by individuals, side effects of ART and other related factors [25]. Additionally, ART cannot eradicate HIV from infected individuals due to the persistence of latent reservoir of HIV-1. Therefore, it is necessary to monitor the HIV nucleic acid to dynamically reflect the therapeutic and provide evidence for adjusting the treatment. Third, the preventive medications, including PrEP (Pre-exposure Prophylaxis) and PEP (Post-exposure Prophylaxis), provide quite effective ways to stop HIV, but if someone has a possible exposures to HIV, taking a quick detection may have impacts on early diagnosis and treatment [26, 27]. Fourth, the imbalance distribution of medical resources in areas affected by HIV epidemic, in some developing countries HIV infected people could not get timely testing or unwilling to get tested. Aimed at the above problems, the CRISPR-based lateral-flow strip assay described in this study provides a powerful visualization tool for on-site HIV-1 RNA with high sensitivity and specificity, minimal equipment, particularly for self-testing at home or in less-developed countries without training staff, biosafety laboratories and equipments [28].

Compared with traditional HIV-1 detection technology, this assay has distinct advantages, the sensitivity of this detection assay for HIV-1 RNA was improved to an LOD of 1 copy/μL, and can be comparable with that of fluorescence CRISPR-based fluorescence assay but higher than the original lateral-flow strip (Commercial strip) and other reported CRISPR-Dx based technology for detecting HIV-1 [15, 24, 29]. Moreover, the ability to rapid detect nucleic acids of the CRISPR-based lateral-flow strip assay also with signal-base specificity. Commercial strips mainly show the detection result by the appearance of T line, the existence of critical value makes it possible to interpret the detection results with some certain subjectivity [15]. In this study, the detection result was shown by the presence or absence of the T line, it’s not affected by the critical value and can be judged more objectively. At present, commercial HIV-1 strip assays mainly focused on antigen or antibody detection, and there is no appropriate HIV-1 RNA strip assay which could meet on-site and point-of-care requirements commercialized [30, 31]. When we tried clinical samples, the lowest detectable viral load was 112copies/mL. First, the sensitivity (1 copy/μL) in this article was the concentration of HIV-1 RNA added to the detection system. If factors such as sample degradation, concentration caused by the nucleic acid extration, addition of sample volume and others are taken into account, the sensitivity of the detection for clinical samples would be even higher. That’s also the reason we could successfully detect clinical samples with the viral load as low as 112 copies/mL. The CRISPR-based lateral-flow strip assay in this study offers a potential HIV-1 early detection technology for resources limited areas that lack training staff and laboratory facilities able to perform molecular diagnostics.

To further evaluation the application effect of the CRISPR-based lateral-flow strip assay in different subtypes of HIV, we used the conserved sites from the major circulating subtype of HIV-1 in China to design crRNA and collected 60 samples covering three known subtypes (CRF07_BC, CRF01_AE and subtype B) for detecting by this assay. Although the detection results of this assay are consistent with RT-qPCR, there is still risk of failing detection due to mutations of the HIV-1 target sequence. When we demonstrate this assay with reasonable sensitivity using crRNAs based on existing PCR primers, we anticipate that in future we could search for the best crRNA combinations. As more information becomes available about viral variants, crRNA design can be adapted to avoid false negative. And a recent study combined crRNAs targeting SARS-CoV-2 RNA to improve the sensitivity and specificity of the CRISPR-Cas13a assay [32]. In future, we will focus more on the emerging HIV-1 variants globally and further optimize the sequence of crRNA to cope with the possible mutations in the target crRNA to avoid false negatives.

HIV testing is usually provided by medical professionals in hospitals, it may also be provided by trained staff from HIV and community health service centers in some countries, self-sampling and self-testing are ways to get tested for HIV at home. In terms of nucleic acid self-testing, this study had several limitations. How to reduce the impact of sampling on the follow-up testing system is considered to be an important factor limiting the development of self-test technology. Therefore, we will optimize the sampling type and develop suitable sample pretreatment technology which can directly release nucleic acid for the purpose of minimizing the impact of sampling on subsequent isothermal amplification and CRISPR detection. In addition, the approach involves two-step RAA amplification and CRISPR reaction, which are slightly complicated. Subsequently, we need to combine the amplification and detection steps into one (One Pot) to further simplify the detection process. Further, integrated with the “line elimination” lateral-flow strip, the CRISPR-based lateral-flow strip assay has the potential to enable rapid, point-of-care home self-testing for HIV.

## Conclusion

In summary, we described a CRISPR-Cas13a-baesd detection methodology for HIV-1 nucleic acid detection using lateral flow assays, our approach offers a promising option for rapid, point-of-care HIV/AIDS testing and a potential technology for promoting early diagnosis and treatment efficacy monitor of HIV patients at home and small clinics.

### Supplementary Information

**Additional file 1: Figure S1.** The HIV-1 RNA template was diluted in gradient and amplified separately using 9 pairs of RT-RAA primers. The amplification products are shown in the red dashed boxes. **Figure S2.** Fluorescence CRISPR detection different concentrations of HIV-1 RNA. Fluorescence CRISPR/Cas13a detection result at 10 min. NC: Negative control, "****" means *P* < 0.0001, "***", "**" means *P* < 0.005, and "ns" means no significant difference. The above experiments were carried out 3 independent repeated experiments. **Figure S3.** Agarose gel electrophoresis of HIV-1 RNA with different concentrations after RT-PCR and RT-RAA. **Figure S4.** RT-RAA-CRISPR/Cas13a detected 158 plasma clinical samples. For fluorescence readings, we set a threshold for the signal-to-noise (S/N) ratio of fluorescence intensity (blue line) (noise is the fluorescence intensity from negative samples performed in parallel with water as input), and positive results are 3. In the lateral-flow strip strip, “T” represents for test bands, and “C” represents for control bands. **Figure S5.** Standard curve for detection of HIV-1 by RT-qPCR. The standard was detected with HIV-1 Nucleic Acid Assay Kit (DaAn Gene Co., Ltd) and a standard curve was drawn. Copy number conversion: 1 IU/mL = 0.51 copy/μL. **Supplementary Table 1.** Sequence information of HIV-1 detected by RT-RAA-CRISPR/Cas13a system. **Supplementary Table 2.** The template sequences used in this study. **Supplementary Table 3.** The crRNA (CRF07_BC, CRF01_AE, B subtype) and primer sequences used in this study.

## Data Availability

The datasets used and analysed during the current study are available from the corresponding author on reasonable request.

## Abbreviations

HIV-1: Human immunodeficiency virus type one
AIDS: Human immunodeficiency virus type one
POCT: Point-of-care testing
ART: Antiretroviral therapy
ELISA: Enzyme-linked immunosorbent assay
COVID-19: Corona Virus Disease 2019
CRISPR-Cas: Clustered regularly interspaced short palindromic repeat and CRISPR-associated protein
SHERLOCK: Specific High Sensitivity Enzymatic Reporter UnLOCKing
DETECTR: DNA Endonuclease-Targeted Crispr Trans Reporter
CDetection: Cas12b-mediated DNA detection
RT-RAA: Reverse transcription recombinase-aided amplification
PCR: Polymerase chain reaction
RT-PCR: Reverse transcription polymerase chain reaction
ERASE: Easy-Readout and Sensitive Enhanced
Cb: Coxiella burnetiid
HBV: Hepatitis B virus
EBOV: Ebola virus
TBEV: Tick-borne encephalitis virus
PrEP: Pre-exposure Prophylaxis
PEP: Post-exposure Prophylaxis
