# Supplementary material for: Highly sensitive and rapid point-of-care testing for HIV-1 infection based on CRISPR-Cas13a system
Source: BMC Infect Dis. 2023 Sep 25;23:627. doi: 10.1186/s12879-023-08492-6 (PMC10518925; doi:10.1186/s12879-023-08492-6)
Supplement: Supplementary file 1 — Additional file 1: Figure S1. The HIV-1 RNA template was diluted in gradient and amplified separately using 9 pairs of RT-RAA primers. The amplification products are shown in the red dashed boxes. Figure S2. Fluorescence CRISPR detection different concentrations of HIV-1 RNA. Fluorescence CRISPR/Cas13a detection result at 10 min. NC: Negative control, "****" means P < 0.0001, "***", "**" means P < 0.005, and "ns" means no significant difference. The above experiments were carried out 3 independent repeated experiments. Figure S3. Agarose gel electrophoresis of HIV-1 RNA with different concentrations after RT-PCR and RT-RAA. Figure S4. RT-RAA-CRISPR/Cas13a detected 158 plasma clinical samples. For fluorescence readings, we set a threshold for the signal-to-noise (S/N) ratio of fluorescence intensity (blue line) (noise is the fluorescence intensity from negative samples performed in parallel with water as input), and positive results are 3. In the lateral-flow strip strip, “T” represents for test bands, and “C” represents for control bands. Figure S5. Standard curve for detection of HIV-1 by RT-qPCR. The standard was detected with HIV-1 Nucleic Acid Assay Kit (DaAn Gene Co., Ltd) and a standard curve was drawn. Copy number conversion: 1 IU/mL = 0.51 copy/μL. Supplementary Table 1. Sequence information of HIV-1 detected by RT-RAA-CRISPR/Cas13a system. Supplementary Table 2. The template sequences used in this study. Supplementary Table 3. The crRNA (CRF07_BC, CRF01_AE, B subtype) and primer sequences used in this study. [file 12879_2023_8492_MOESM1_ESM.docx]

***Supplementary Material***

1. **Supplementary Figures and Tables**
   1. **Supplementary Figures**

#
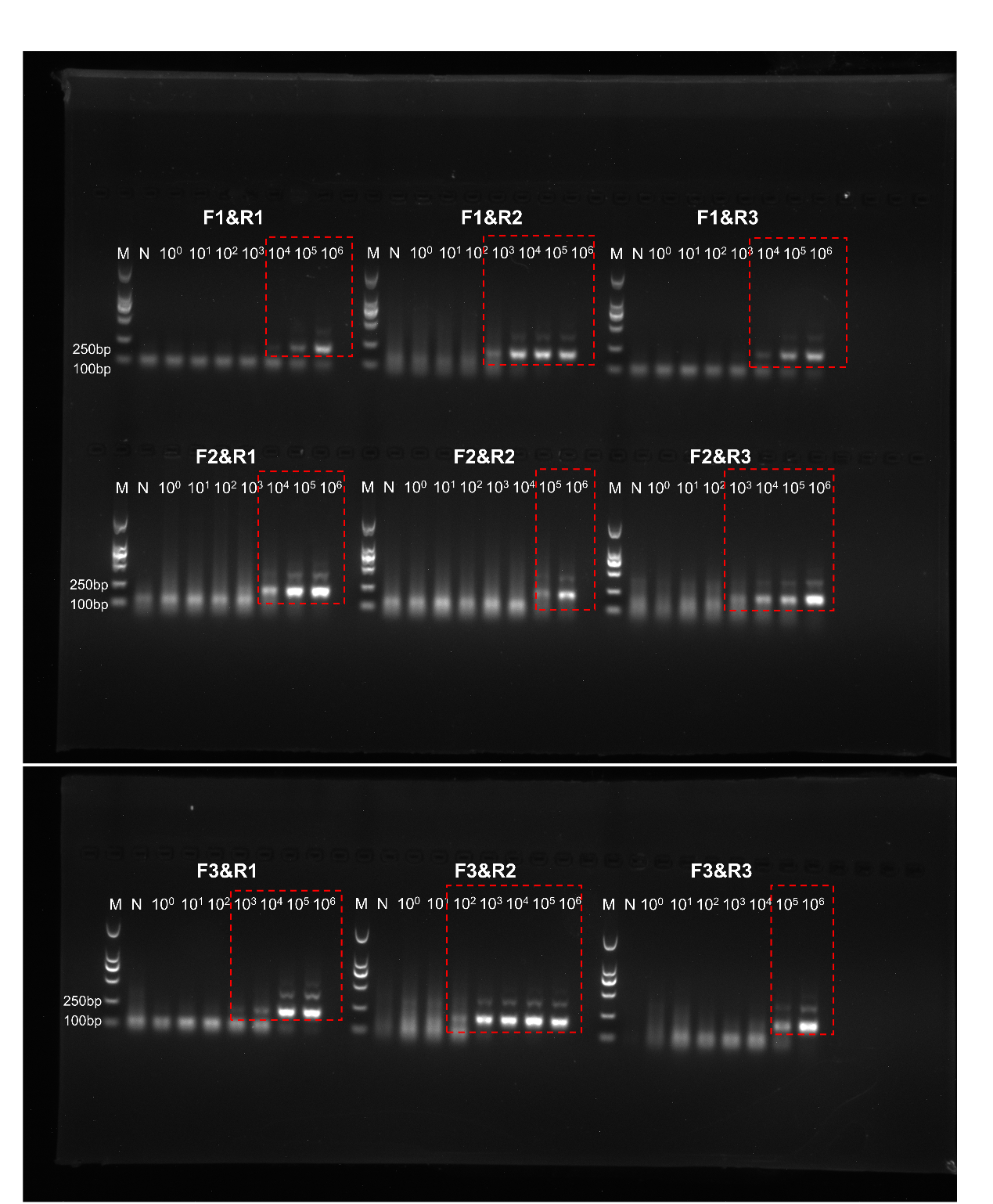


**Figure S1.** The HIV-1 RNA template was diluted in gradient and amplified separately using 9 pairs of RT-RAA primers. The amplification products are shown in the red dashed boxes

**
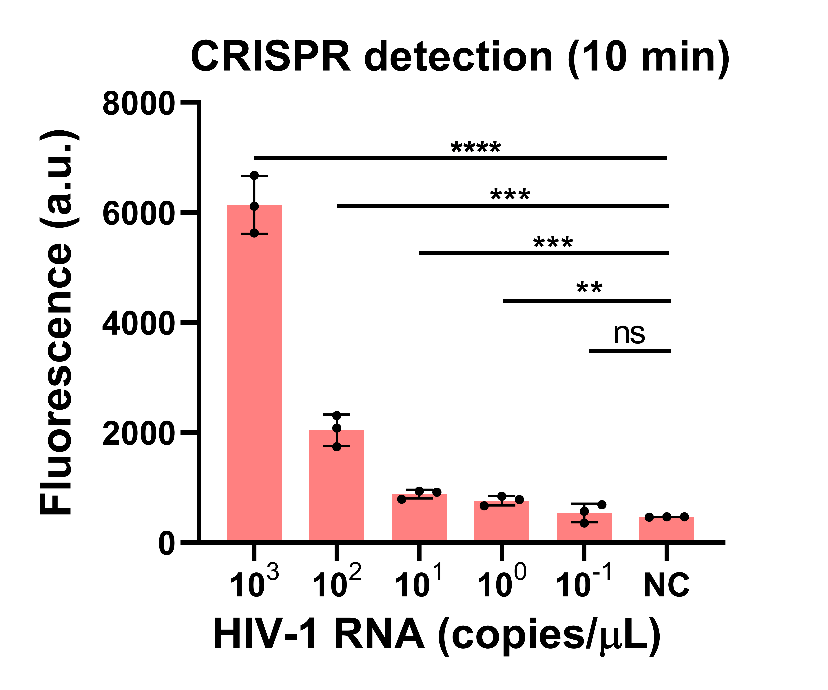
**

**Figure S2.** Fluorescence CRISPR detection different concentrations of HIV-1 RNA. Fluorescence CRISPR/Cas13a detection result at 10 min. NC: Negative control, "****" means P < 0.0001, "***", "**" means P < 0.005, and "ns" means no significant difference. The above experiments were carried out 3 independent repeated experiments.


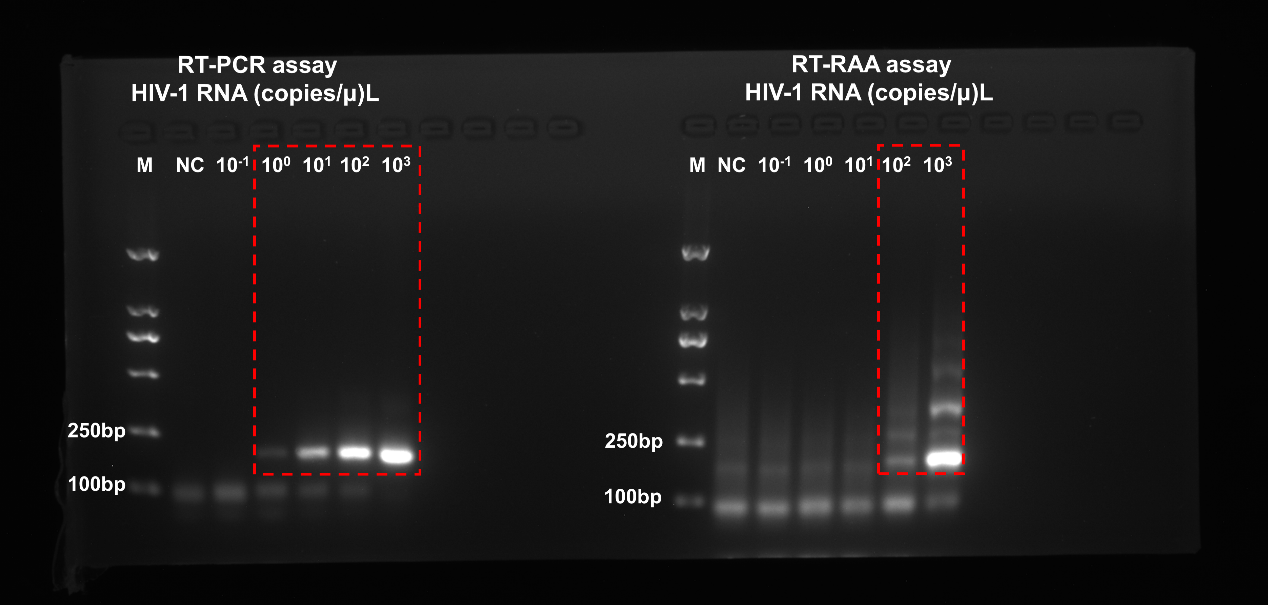


**Figure S3.** Agarose gel electrophoresis of HIV-1 RNA with different concentrations after RT-PCR and RT-RAA


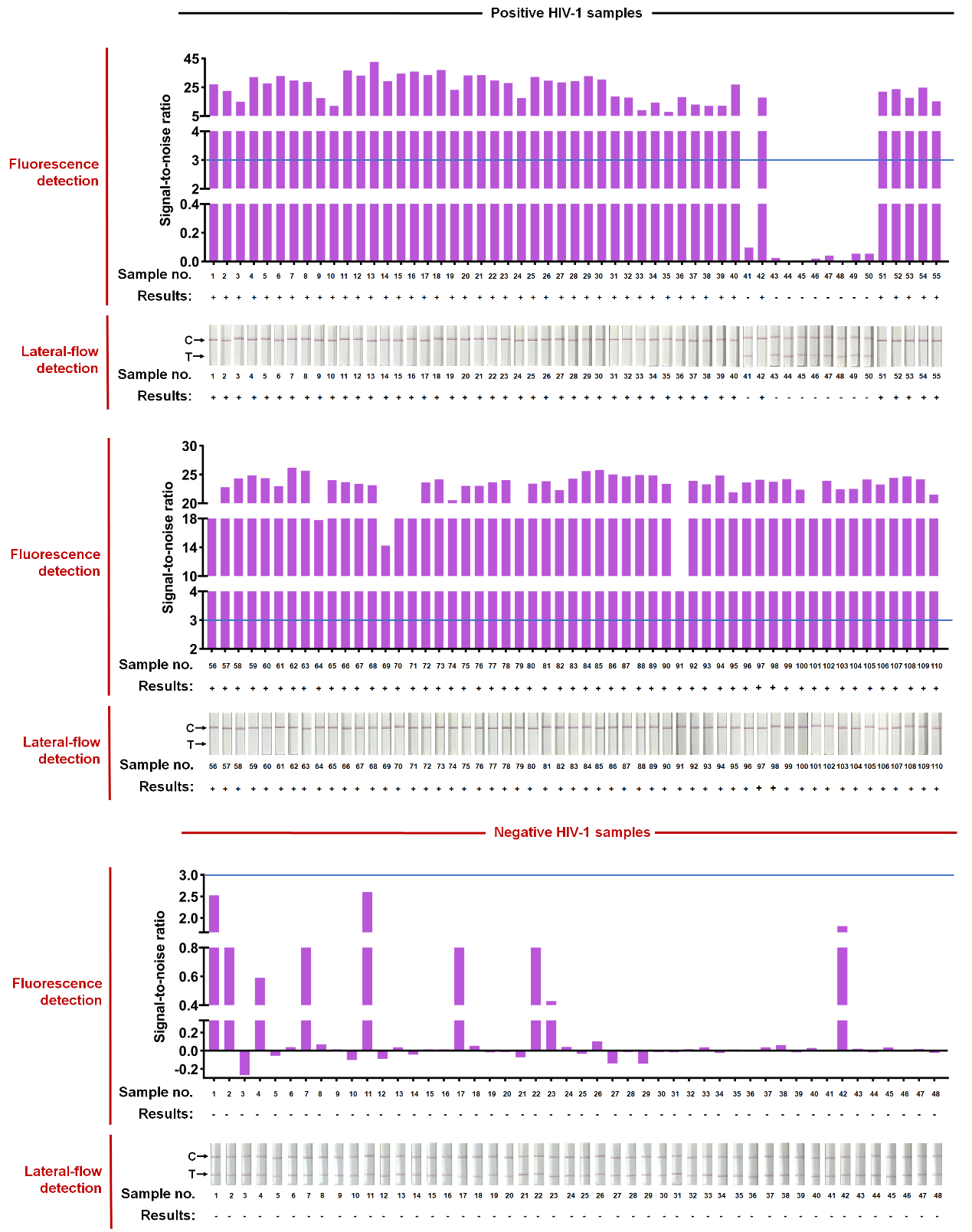


**Figure S4.** RT-RAA-CRISPR/Cas13a detected 158 plasma clinical samples. For fluorescence readings, we set a threshold for the signal-to-noise (S/N) ratio of fluorescence intensity (blue line) (noise is the fluorescence intensity from negative samples performed in parallel with water as input), and positive results are 3. In the lateral-flow strip strip, “T” represents for test bands, and “C” represents for control bands.

**
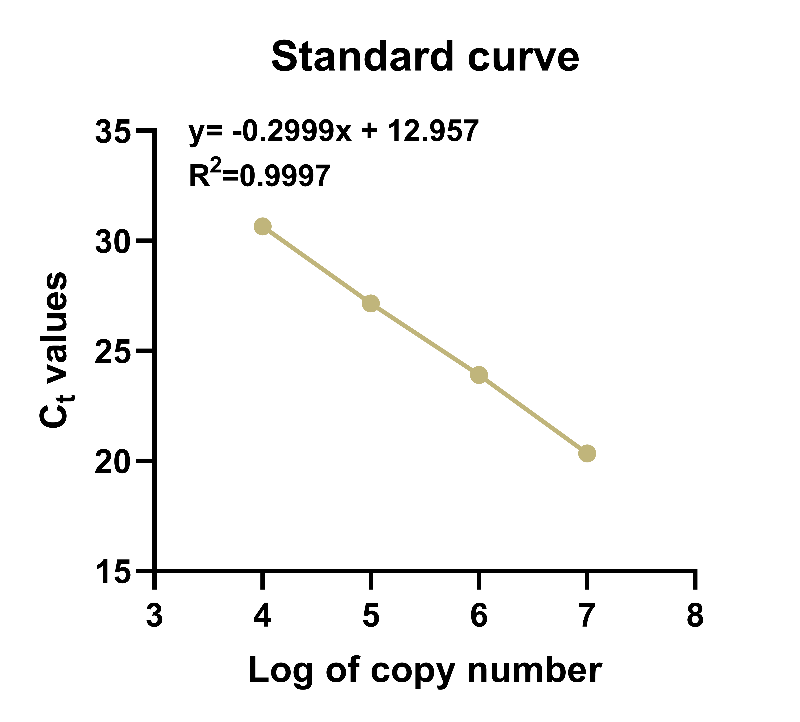
**

**Figure S5.** Standard curve for detection of HIV-1 by RT-qPCR. The standard was detected with HIV-1 Nucleic Acid Assay Kit (DaAn Gene Co., Ltd) and a standard curve was drawn. Copy number conversion: 1 IU/mL = 0.51 copy/μL.

- 1. **Supplementary Tables**

**Supplementary Table 1** Sequence information of HIV-1 detected by RT-RAA-CRISPR/Cas13a system

| **Name** | **Sequence（5’- 3’）** |
| --- | --- |
| **HIV-crRNA-F** | taatacgactcactatagGGGATTTAGACTACCCCAA |
| **HIV-crRNA1** | GGGATTTAGACTACCCCAAAAACGAAGGGGACTAAAACATTCTTTCCCCTGCACTGTACCCCCCAA |
| **HIV-crRNA-R1** | ttggggggtacagtgcaggg |
| **HIV-crRNA2** | GGGATTTAGACTACCCCAAAAACGAAGGGGACTAAAACATGTCTACTATTCTTTCCCCTGCACTGT |
| **HIV-crRNA-R2** | acagtgcaggggaaagaata |
| **HIV-crRNA3** | GGGATTTAGACTACCCCAAAAACGAAGGGGACTAAAACTTATGTCTACTATTCTTTCCCCTGCACT |
| **HIV-crRNA-R3** | agtgcaggggaaagaatagt |
| **HIV-crRNA4** | GGGATTTAGACTACCCCAAAAACGAAGGGGACTAAAACTGCTATTATGTCTACTATTCTTTCCCCT |
| **HIV-crRNA-R4** | aggggaaagaatagtagaca |
| **RT-RAA-F1** | AATTCTAATACGACTCACTATAGGGCTTAAGACAGCAGTACAAATGGCAGTATTC |
| **RT-RAA-F2** | AATTCTAATACGACTCACTATAGGGCTTAARACAGCAGTACARATGGCAGTATTCAT |
| **RT-RAA-F3** | AATTCTAATACGACTCACTATAGGGcagcagtacaaatggcagtattcatYcaca |
| **RT-RAA-R1** | TCTCTGCTGTCRCTGTAATAAACCCGAAAATTTTG |
| **RT-RAA-R2** | GTCRCTGTAATAAACCCGAAAATTTTGAATTT |
| **RT-RAA-R3** | TCTGCTGTCRCTGTAATAAACCCGAAAATTTTG |
| **RT-PCR-F** | TAAGACAGCAGTACAAATGGC |
| **RT-PCR-R** | TCTGCTGTCRCTGTAATAAAC |
| **Report RNA** | FAM-UUUUUUUUUUUUUUUUUUUU-Biotin |

**Supplementary Table 2** The template sequences used in this study.

| **Name** | **Sequences (5’-3’)** |
| --- | --- |
| pol | attccctacaatccccaaagtcaaggagtagtagaatctatgaataaagaattaaagaaaattataggacaggtaagagatcaggctgaacatcttaagacagcagtacaaatggcagtattcatccacaattttaaaagaaaaggggggattggggggtacagtgcaggggaaagaatagtagacataatagcaacagacatacaaactaaagaattacaaaaacaaattacaaaaattcaaaattttcgggtttattacagggacagcaga |

**Supplementary Table 3** The crRNA (CRF07_BC, CRF01_AE, B subtype) and primer sequences used in this study.

| **Name** | **Sequence（5’- 3’）** |
| --- | --- |
| **crRNA-F** | taatacgactcactatagGGGATTTAGACTACCCCAA |
| **crRNA** | GGGATTTAGACTACCCCAAAAACGAAGGGGACTAAAACTTATRTCTATTATTCTTTCYCCTGCACT |
| **crRNA-R** | AGTGCAGGRGAAAGAATAAT |
| **RT-RAA-F** | AATTCTAATACGACTCACTATAGGG cttaagacagcagtacaaatggcagtatt |
| **RT-RAA-R** | GTAYTTCTTTAGTTTGTATGTCTGWTGCTA |
